# Reducing Periprosthetic Joint Infection in Patients With Obesity: A Systematic Review and Meta-Analysis of the Emerging Role of Glucagon-Like Peptide-1 (GLP-1) Receptor Agonists

**DOI:** 10.7759/cureus.99805

**Published:** 2025-12-21

**Authors:** André Fernandes, Charlotte MacAulay, Abdul R Khater, Hannah Matthews, Ganesh Mohrir, Christopher Lodge

**Affiliations:** 1 Trauma and Orthopaedics, York and Scarborough Teaching Hospitals NHS Foundation Trust, York, GBR

**Keywords:** arthroplasty, glp-1 receptor agonist, meta-analysis, obesity, periprosthetic joint infection, semaglutide, systematic review, tirzepatide

## Abstract

Obesity is a well-established risk factor for perioperative morbidity, including periprosthetic joint infection (PJI) and unplanned readmission following total hip and knee arthroplasty. Glucagon-like peptide-1 (GLP-1) receptor agonists induce clinically meaningful weight loss and metabolic optimisation, yet their perioperative influence in arthroplasty populations remains uncertain. In this systematic review and meta-analysis of observational cohorts comprising adults with obesity undergoing primary hip or knee arthroplasty, outcomes were compared between patients receiving perioperative GLP-1 therapy and those not exposed to these agents. Eight cohorts encompassing 95,503 individuals, including 22,098 GLP-1 users, met inclusion criteria. Perioperative GLP-1 therapy was associated with a significantly reduced incidence of PJI after total hip arthroplasty and fewer 90-day hospital readmissions, corresponding to an absolute risk reduction of approximately 1.8% from a median baseline risk of 9% and an estimated number needed to treat of 56. No significant association was demonstrated for PJI following total knee arthroplasty or for revision procedures, and GLP-1 therapy was not linked to increased rates of venous thromboembolism, acute kidney injury, cerebrovascular events, myocardial infarction, or hypoglycaemia. Certainty of evidence was moderate for hip PJI and readmission and low for knee PJI and revision outcomes. These findings suggest that perioperative GLP-1 therapy may confer clinically relevant benefits in selected arthroplasty populations, underscoring the need for well-designed randomised trials to define optimal perioperative protocols, economic value, and long-term implant survivorship.

## Introduction and background

Total knee arthroplasty (TKA) and total hip arthroplasty (THA) are among the most successful orthopaedic procedures, with over 220,000 performed annually in the United Kingdom (UK) [1]. Obesity increases perioperative risks, including wound complications, periprosthetic joint infection (PJI), and hospital readmission [2]. Approximately 28% of adults in the UK live with obesity; among arthroplasty candidates, prevalence approaches 40%, contributing disproportionately to complications and healthcare costs [3,4].

Conventional optimisation strategies (e.g., bariatric surgery and dietetic programmes) are limited by low uptake, long waiting times, and inconsistent clinical benefit. Prior studies and reviews have reported no consistent reduction in complications after bariatric surgery, with some analyses suggesting increased early dislocation and mortality [5,6], potentially related to nutrient malabsorption, impaired bone metabolism, and altered pharmacokinetics [7].

Glucagon-like peptide-1 (GLP-1) receptor agonists, initially indicated for type 2 diabetes, obesity care by reducing appetite and improving satiety without nutrient malabsorption. Large phase III trials report 15-20% mean weight loss, together with improvements in glycaemic control and inflammatory markers. These findings support the evaluation of GLP-1 therapy in the perioperative arthroplasty setting [8-10].

To our knowledge, no prior systematic review and meta-analysis has synthesised perioperative GLP-1 effects in arthroplasty. We therefore evaluated whether GLP-1 therapy reduces PJI, revision, and early readmission in adults suffering with obesity undergoing THA or TKA.

## Review

Methods

This systematic review and meta-analysis followed the Cochrane Handbook for Systematic Reviews of Interventions [11] and Preferred Reporting Items for Systematic Reviews and Meta-Analyses (PRISMA) 2020 reporting guidance [12]. The protocol was prospectively registered on the International Prospective Register of Systematic Reviews (PROSPERO) (CRD420251108739).

Eligibility Criteria

**Figure 1:**
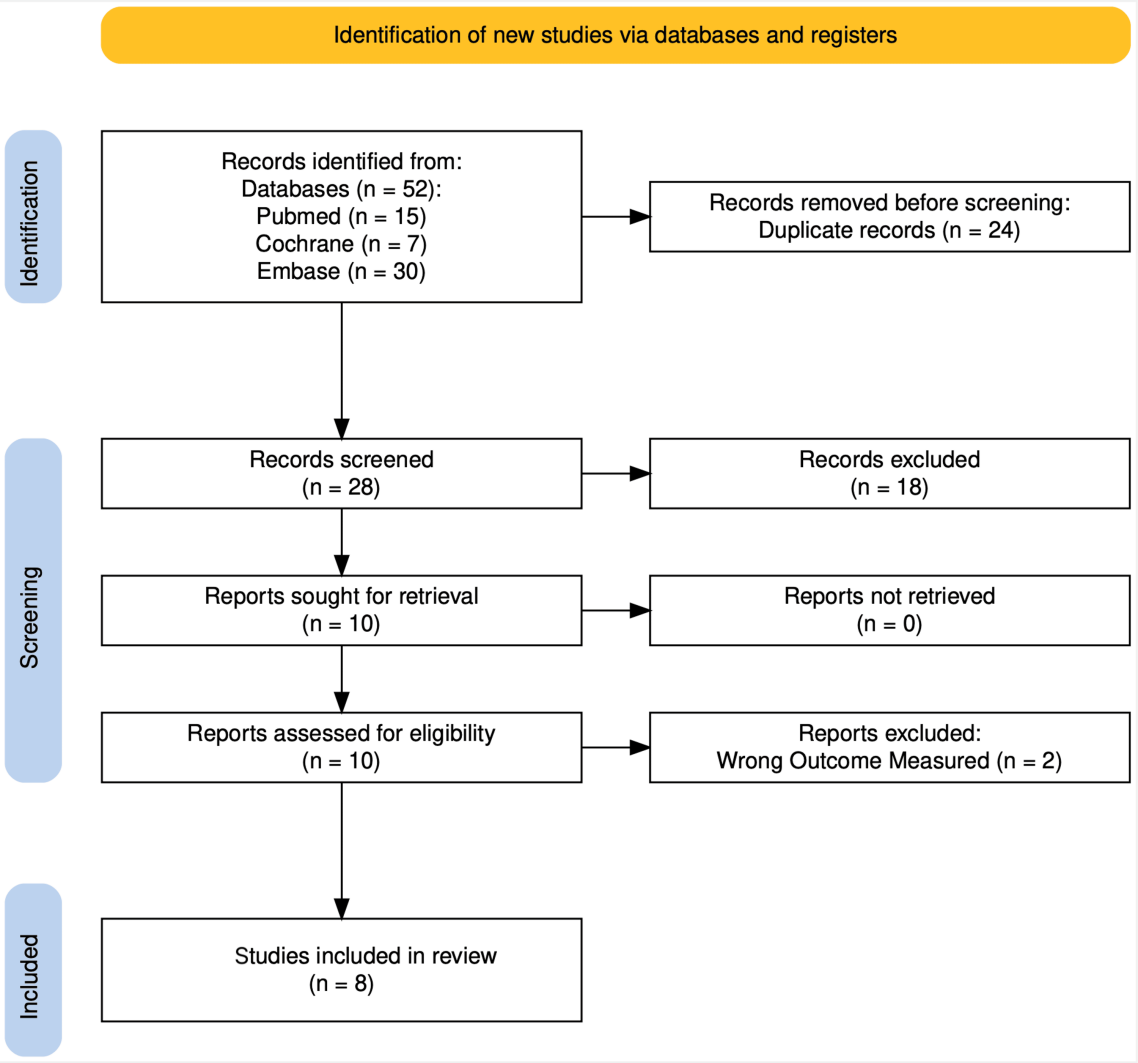
PRISMA flow diagram. PRISMA: Preferred Reporting Items for Systematic Reviews and Meta-Analyses

We included observational comparative studies of adults with obesity undergoing primary THA or TKA. For the purposes of this review, our primary population of interest was defined as BMI ≥30 kg/m². In studies that reported mixed BMI categories, we included only cohorts or subgroups meeting this BMI threshold. Where original articles explicitly defined their arthroplasty population as “overweight/obesity with comorbidities” using a BMI cut-off of ≥27 kg/m² plus weight-related comorbidities, these data were extracted and considered in prespecified sensitivity analyses and are clearly indicated as such in the Results. Eligible studies evaluated perioperative use of a GLP-1 receptor agonist (semaglutide, liraglutide, exenatide, dulaglutide, or tirzepatide) compared with no GLP-1 therapy, with ≥12 months’ follow-up and at least one prespecified outcome. We excluded trauma cases, revision or oncological arthroplasty, studies not published in English, and all non-original research (Figure 1).

The primary outcomes were PJI and revision surgery. Secondary outcomes included hospital readmission, venous thromboembolism (deep vein thrombosis or pulmonary embolism), acute kidney injury, cerebrovascular accident, myocardial infarction, and hypoglycaemia.

Search Strategy

**Figure 2:**
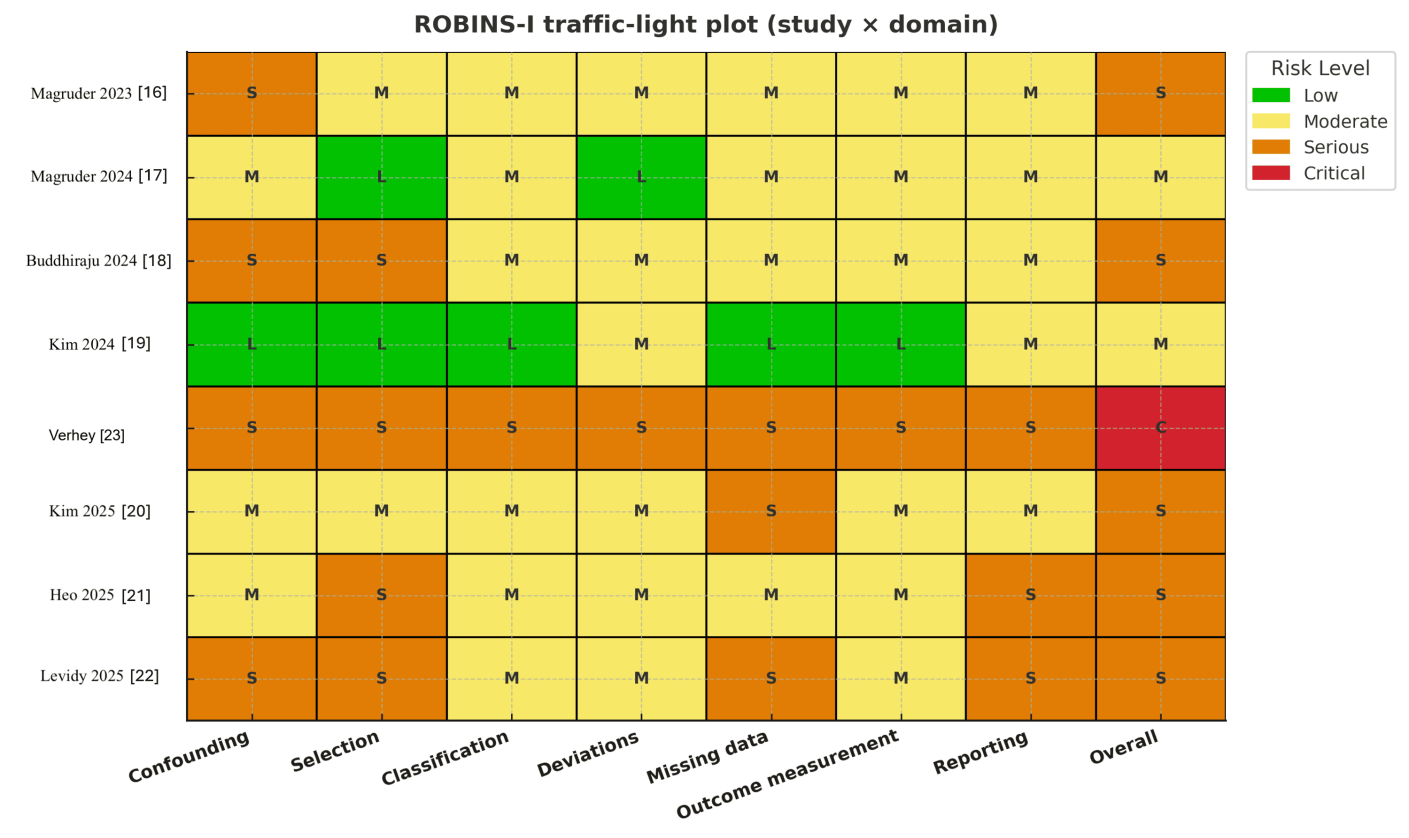
ROBINS-I traffic-light plot Traffic-light summary of ROBINS-I risk-of-bias assessments for each included study across individual domains and overall risk. Colours correspond to standard ROBINS-I categories: green = low risk, yellow = moderate risk, orange = serious risk, red = critical risk. Study identifiers are listed on the y-axis. ROBINS-I: Risk Of Bias In Non-randomised Studies - of Interventions

**Figure 3:**
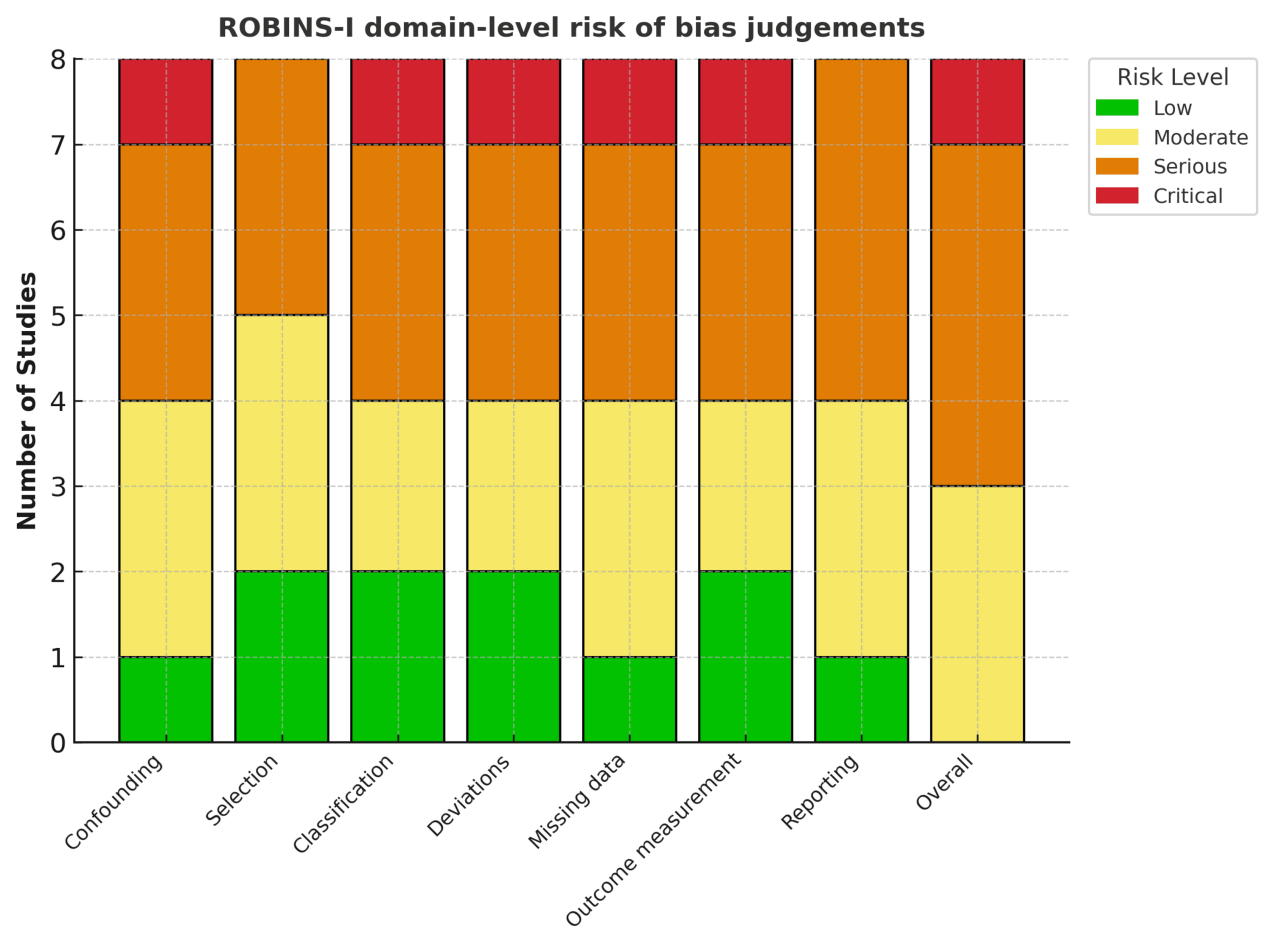
ROBINS-I domain-level summary. Stacked bar chart showing the distribution of ROBINS-I domain-level risk-of-bias judgements across all included studies. Each bar represents a domain, with colours denoting the number of studies judged at low (green), moderate (yellow), serious (orange), or critical (red) risk of bias. ROBINS-I: Risk Of Bias In Non-randomised Studies - of Interventions

A systematic search of PubMed, Embase, and the Cochrane Library was performed on June 24, 2025. Reference lists of included articles and relevant reviews were also screened. Two reviewers independently screened titles, abstracts, and full texts, with disagreements resolved by consensus or senior author review. Data were extracted independently, and the risk of bias was assessed using the ROBINS-I (Risk Of Bias In Non-randomized Studies - of Interventions) tool for non-randomised studies (Figures 2, 3) [13]. The certainty of evidence for each outcome was appraised using GRADE (Grading of Recommendations Assessment, Development, and Evaluation) methodology [14].

Study-level odds ratios (ORs) were pooled on the log scale using an inverse-variance random-effects model. Between-study variance (τ²) was estimated by restricted maximum likelihood (REML). Wald-type 95% confidence intervals (CIs) were calculated for pooled and subgroup effects. Heterogeneity was assessed with Cochran’s Q, I², H², and τ². Prespecified sensitivity analyses included leave-one-out influence analysis and restriction to studies at low or moderate risk of bias (ROBINS-I). For clinical interpretability, illustrative absolute risk differences were derived from the pooled OR and the median control risk. Analyses were performed in Review Manager (RevMan) Web 5.4 (2020; Cochrane Collaboration, London, UK) [15]. We selected a random-effects framework a priori because populations, GLP-1 agents/exposure windows, and adjustment sets varied across studies. *Data Collection and Cohort Definition* For each eligible study, two reviewers independently extracted data using a standardised form, with a prespecified focus on patients with obesity. Wherever possible, we restricted extraction to adults with obesity, defined as BMI ≥30 kg/m². When primary cohorts included both obese and non-obese patients, we extracted only the subgroup data that met this BMI criterion (e.g., stratified results for BMI ≥30 kg/m², ≥35 kg/m², or ≥40 kg/m²). In a minority of studies that explicitly defined their arthroplasty population as overweight or obese with comorbidities using a BMI cut-off of ≥27 kg/m², these cohorts were extracted separately and included in prespecified sensitivity analyses; they are clearly labelled in the Results. If obese-specific data could not be separated from all-comer cohorts, the study was excluded from the primary meta-analyses and, where appropriate, considered only in sensitivity analyses that are clearly labelled as such. For all included obese cohorts, we extracted sample size, BMI criteria, GLP-1 agent, and exposure window, comparator definition, follow-up duration, and outcome-specific event counts or adjusted effect estimates, thereby aligning the analytic dataset with our central hypothesis that perioperative GLP-1 therapy reduces periprosthetic joint infection in adults with obesity.

Risk of Bias and Certainty of Evidence

Two studies were at moderate risk [16,17], five at serious risk [18-22], and one at critical risk [23]. Common concerns were residual confounding (e.g., metabolic comorbidities and perioperative optimisation), selection bias (including potential immortal-time/prevalent-user bias), and missing data. Outcome definitions were generally consistent across studies.

**Figure 4:**
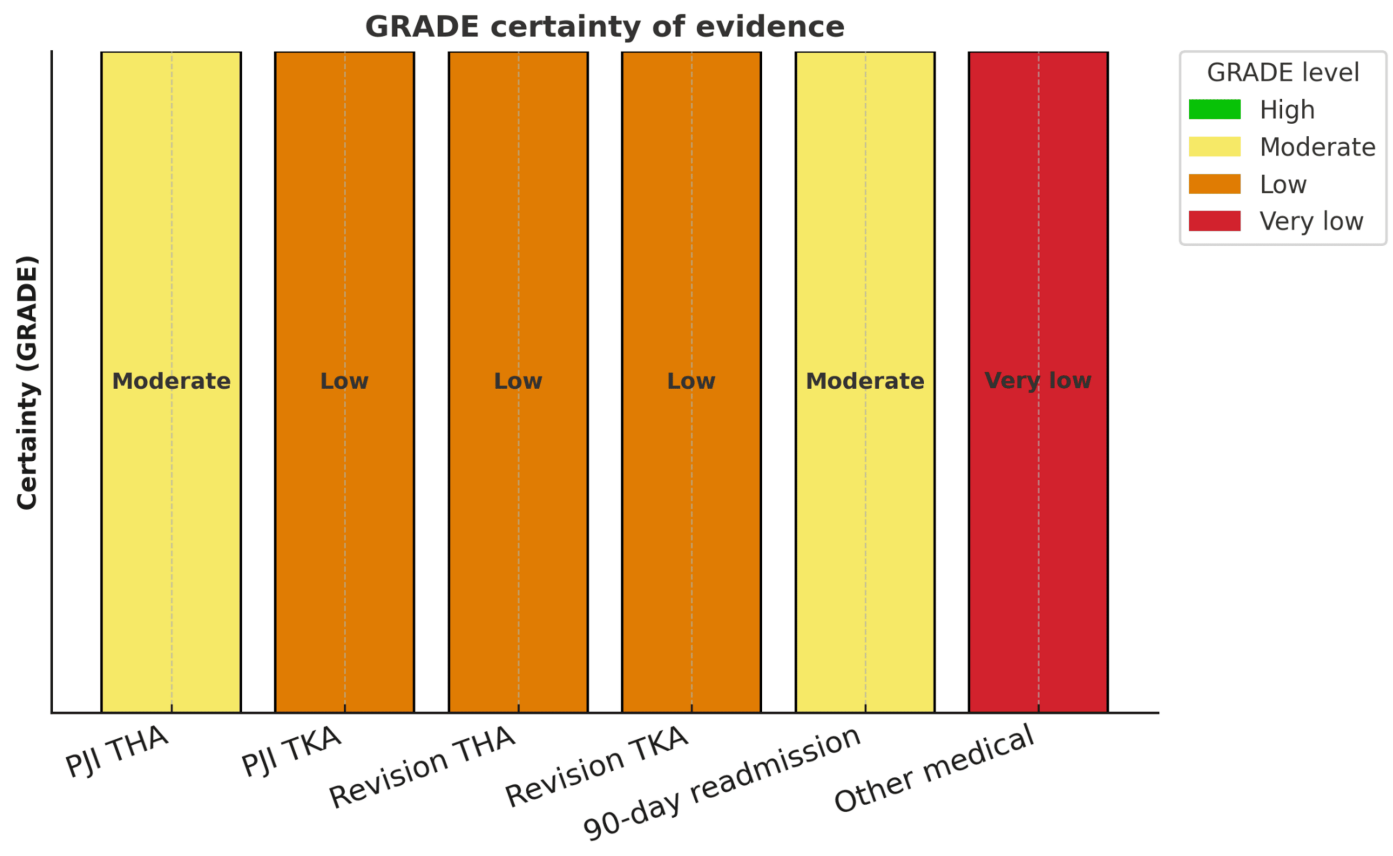
GRADE certainty of evidence by outcome. Certainty of evidence appraised using the GRADE framework for each prespecified outcome. Bars indicate the overall certainty rating (very low, low, moderate, high) based on study design, risk of bias, inconsistency, indirectness, imprecision, and publication bias. Colours follow the standard GRADE/traffic-light convention: red = very low, orange = low, yellow = moderate, green = high. PJI: periprosthetic joint infection; THA: total hip arthroplasty; TKA: total knee arthroplasty; GRADE: Grading of Recommendations Assessment, Development, and Evaluation

Integrating ROBINS-I with GRADE, certainty was moderate for THA-PJI and 90-day readmission, low for TKA-PJI and revision, and very low for infrequent medical complications, reflecting retrospective designs, incomplete confounder adjustment, and heterogeneity despite consistent effect directionality (Figure 4). We also judged indirectness for some outcomes to be present due to residual variability in BMI thresholds across source studies (e.g., differing definitions of severe and morbid obesity), which may contribute to clinical heterogeneity despite our efforts to standardise the primary analytic population to BMI ≥30 kg/m².

Results

Our search identified 52 records, of which 24 duplicates were removed. After screening 28 abstracts and reviewing 10 full texts, eight retrospective cohort studies met eligibility criteria and were included in the analysis (Figure 1). Collectively, these studies reported on 95,503 patients with obesity (or overweight with comorbidities where explicitly defined), including 22,098 who received perioperative GLP-1 therapy and 73,405 controls. For the purposes of this review, perioperative GLP-1 therapy was defined as GLP-1 receptor agonist use initiated and/or continued during the preoperative period (typically up to ≥3 months before arthroplasty) and extending into the early postoperative phase, according to each study’s specified exposure window. Four studies used large national or insurance databases, while the remaining four were institutional cohorts. Semaglutide was the most frequently reported GLP-1 agent. The mean follow-up ranged from 12 to 36 months (Table 1).

**Table 1:** Characteristics of the studies included in the meta-analysis ^a^ Severe obesity includes individuals with a BMI of 35.0 to 39.9 kg/m²; ^b^ Morbid obesity includes individuals with BMI ≥40 kg/m² NA: not available; SO: severe obesity; MO: morbid obesity; TKA: total knee replacement; THA: total hip replacement

Study	Follow-Up (months)	Total Patients	Number of patients				Diabetic Patients, n				Overweight or Patients with Obesity, n				Age (years), mean/mean (SD)		Male Sex, n (%)	
			TKA		THA		TKA		THA		TKA		THA		GLP1	Control	GLP1	Control
			GLP1	Control	GLP1	Control	GLP1	Control	GLP1	Control	GLP1	Control	GLP1	Control				
Magruder et al., 2023 [16]	24	41575	7510	34524	0	0	7051	34524	0	0	6081	29844	0	0	62.4	62.5	2720 (38.6)	13265 (38.4)
Magruder et al., 2024 [17]	23	9465	0	0	1653	7812	0	0	1653	7812	0	0	1404	6655	61.91	62.4	866 (52.4)	4076 (52.2)
Kim et al., 2025 [20]	3 + 24	11899	2975	8924	0	0	1571	4721	0	0	2975	8924	0	0	62.2 (7.6)	SO: 62.3 (7.6); MO: 62.2 (7.6)	989 (33.2)	SO: 1982 (33.3), MO: 986 (33.1)
Heo et al., 2025 [21]	3 + 12	4060	0	0	812	3248	0	0	812	3248	0	0	NA	NA	61	60	473 (58.3)	1871 (57.6)
Levidy et al., 2025 [22]	3 + 21	9400	4700	4700	2244	2244	4700	4700	2244	2244	NA	NA	NA	NA	NA	NA	NA	NA
Verhey et al., 2025 [23]	24	10690	0	0	5345	5345	0	0	0	0	0	0	3650	3647	57 (8.6)	57 (8.5)	1671 (45.48)	1656 (44.91)
Kim et al., 2024 [19]	3 + 24	6939	0	0	771	6168	0	0	403	3217	0	0	771	6168	62.1 (8.3)	SO: 62.1 (8.5); MO: 62.1 (8.3)	364 (47.2)	SO: 1484 (48.1), MO: 1453 (47.1)
Buddhiraju et al., 2024 [18]	3	3139	2095	2095	1044	1044	1452	1432	717	714	1473	1535	718	737	THA: 63.3 TKA: 64.1	THA: 42.3; TKA: 32.6	NA	NA

Periprosthetic Joint Infection - TKA

**Figure 5:**
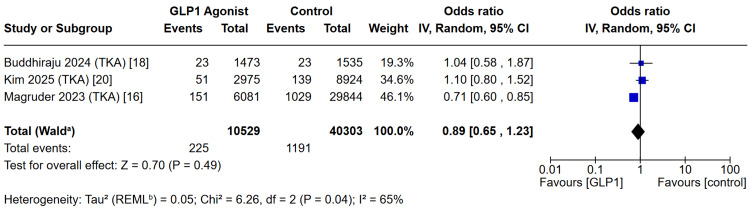
Forest plot for primary endpoint: PJI in the TKA cohort ^a^ CI calculated by Wald-type method; ^b^ Tau^2^ calculated by restricted maximum likelihood method PJI: periprosthetic joint infection; TKA: total knee arthroplasty; REML: restricted maximum likelihood References: [16,18,20]

Three studies contributed to the meta-analysis for PJI following TKA, comprising 50,832 patients in total, including 10,529 GLP-1 users and 40,303 controls [16,18,20]. Across these studies, there was no significant association between perioperative GLP-1 therapy and PJI after TKA (pooled OR 0.89, 95%CI 0.65-1.23; Z = 0.70, p = 0.49). Between-study heterogeneity was moderate to substantial (τ² = 0.05; p = 0.04; I² = 65%) (Figure 5).

Periprosthetic Joint Infection - THA

**Figure 6:**
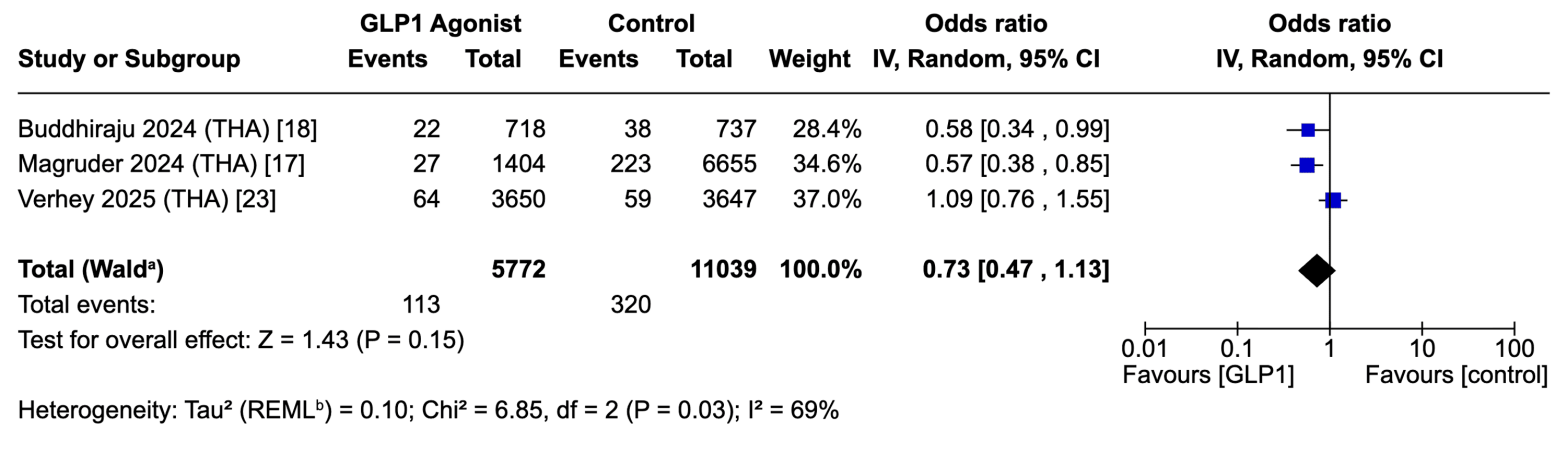
Forest plot for primary endpoint: PJI in the THA cohort. ^a^ CI calculated by Wald-type method; ^b^ Tau^2^ calculated by restricted maximum likelihood method PJI: periprosthetic joint infection; THA: total hip arthroplasty; REML: restricted maximum likelihood References: [17,18,23]

Three studies contributed to the meta-analysis for PJI following THA, comprising 16,811 patients in total, including 5,772 GLP-1 users and 11,039 controls [17,18,23]. Perioperative GLP-1 therapy was associated with a non-significant trend toward reduced PJI after THA (pooled OR 0.73, 95%CI 0.47-1.13; Z = 1.43, p = 0.15). Between-study heterogeneity was substantial (τ² = 0.10; p = 0.03; I² = 69%) (Figure 6).

Revision Surgery - TKA

**Figure 7:**
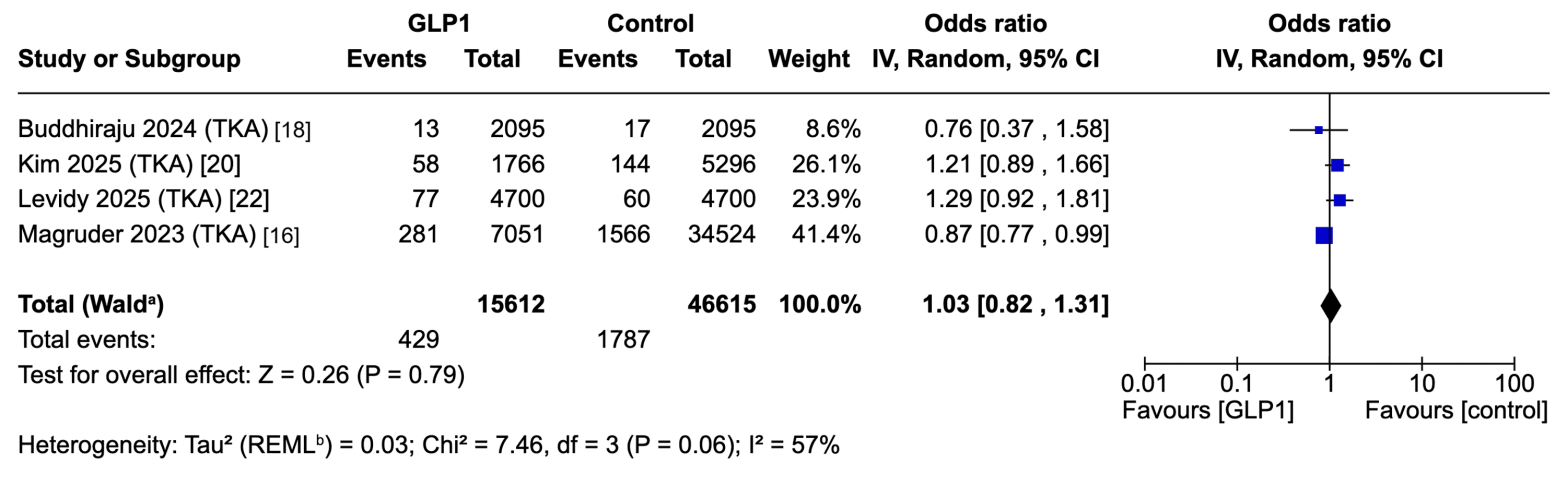
Forest plot for primary endpoint: revision surgery in TKA cohort. ^a^ CI calculated by Wald-type method; ^b^ Tau^2^ calculated by restricted maximum likelihood method PJI: periprosthetic joint infection; TKA: total knee arthroplasty; REML: restricted maximum likelihood Reference: [16,18,20,22]

Four studies (62,227 patients) found no difference in revision following TKA (OR 1.03, 95%CI 0.82-1.31; p = 0.79), with moderate heterogeneity (I² = 57%, τ² = 0.03) (Figure 7) [16,18,20,22].

Revision Surgery - THA

**Figure 8:**
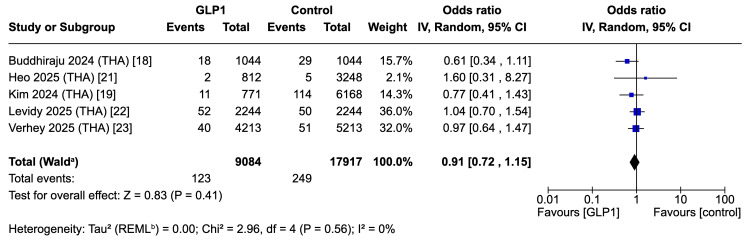
Forest plot for primary endpoint: revision surgery in THA cohort ^a^ CI calculated by Wald-type method; ^b^ Tau^2^ calculated by restricted maximum likelihood method THA: total hip arthroplasty; REML: restricted maximum likelihood References: [18,19,21-23]

Five studies (36,466 patients) reported no difference in revision risk after THA between GLP-1 and control groups (OR 0.91, 95% CI 0.72-1.15; p = 0.41). Heterogeneity was negligible (I² = 0%, τ² = 0.00) (Figure 8) [18,19,21-23].

Secondary Outcomes

**Figure 9:**
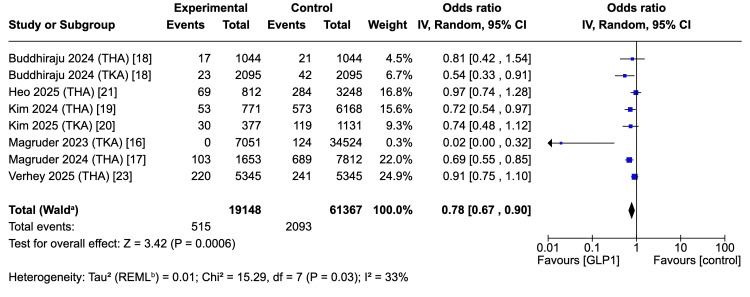
Forest plot: readmission ^a^ CI calculated by Wald-type method; ^b^ Tau^2^ calculated by restricted maximum likelihood method. NOTE: The two different sub-cohorts (THA and TKA) of Buddhiraju et al., 2024 [18] are treated as two different studies. THA: total hip arthroplasty; TKA: total knee arthroplasty; REML: restricted maximum likelihood References: [16-21,23]

Seven studies comprising 95,503 patients (22,098 GLP-1 users) demonstrated a significant reduction in 90-day readmissions with GLP-1 therapy (OR 0.78, 95%CI 0.67-0.90; p < 0.001) [16-21,23]. Heterogeneity was low-to-moderate (I² = 33%, τ² = 0.01). This equates to an absolute risk reduction of ~1.8% from a median baseline risk of 9%, corresponding to a number needed to treat (NNT) of ~56 (Figure 9).

In contrast, no significant differences were observed in the incidence of venous thromboembolism, acute kidney injury, cerebrovascular accident, or myocardial infarction. These complications were infrequent in both groups, and pooled estimates showed no signal of harm. This finding is consistent with large cardiovascular outcome trials of GLP-1 receptor agonists, which collectively demonstrate cardiovascular safety and, in several cases, reductions in major adverse cardiovascular events [23-25]. Meta-analyses further confirm a neutral to favourable thromboembolic and renal risk profile, supporting the overall perioperative safety of these agents [26,27].

Discussion

In a total population of over 95,000 arthroplasty patients suffering from obesity, perioperative GLP-1 therapy was associated with fewer PJIs after THA (OR 0.74) and lower 90-day readmissions (OR 0.78). No significant effects were seen for TKA-PJI or revision. The THA signal was robust to sensitivity analyses, whereas the TKA analysis exhibited substantial heterogeneity and sensitivity to single-study influence.

These findings are consistent with prior observational cohorts reporting lower infection and readmission rates among patients receiving perioperative GLP-1 therapy [15-22]. Although evidence remains observational with residual confounding, reproducibility across heterogeneous datasets suggests potential utility as a perioperative optimisation tool.

In contrast, bariatric surgery has not consistently reduced arthroplasty complications and, in some analyses, has been associated with higher early dislocation or mortality [5-7]. Mechanisms may include malabsorption, altered bone metabolism, and pharmacokinetics. GLP-1 receptor agonists offer metabolic optimisation without malabsorption, supporting their pragmatic perioperative use [8,9].

Morbid obesity (BMI ≥40 kg/m²) may modify the effect of GLP-1 therapy. Across database-driven cohorts focusing on patients with morbid obesity, greater absolute reductions in early complications and readmissions have been reported [18,19,21-23]. However, estimates vary with exposure window (e.g., ≥3 months pre-op and continuation postoperatively), agent selection, and adjustment sets. These subgroup observations should be interpreted cautiously and confirmed prospectively.

Revision outcomes likely require longer follow-up to detect differences; current cohorts (12-36 months) may be underpowered for implant-survivorship endpoints. Findings from broad anti-obesity medication analyses are consistent with our null revision meta-analysis but were not included due to differing exposure definitions [28,29].

Limitations

All included studies were retrospective and observational in design and therefore vulnerable to residual confounding despite statistical adjustment. Confounding by indication is likely, as patients receiving GLP-1 receptor agonists may differ systematically from controls in metabolic optimisation, healthcare utilisation, and access to perioperative care pathways. Selection bias, including prevalent user and immortal time bias, may also have arisen from heterogeneity in exposure definitions. Outcome misclassification remains possible because administrative coding was used to identify periprosthetic joint infection, revision, and medical complications.

Although our primary analyses were restricted to adults with obesity defined as a BMI of at least 30 kg per square metre, variability in obesity thresholds across source studies introduces clinical heterogeneity and indirectness. Differences in the categorisation of severe and morbid obesity and occasional inclusion of individuals with a BMI of at least 27 kg/m^2^ with comorbidities limit uniformity of the analytic population. In several cohorts, obese specific subgroup data could not be fully separated from all-comer populations and were therefore excluded from primary analyses or included only in sensitivity analyses. Some degree of population misclassification, therefore, remains possible.

Definitions of perioperative GLP-1 exposure varied substantially between studies with respect to timing, duration, and continuation into the postoperative period, which limits causal interpretation and precludes dose-response analysis. Heterogeneity in GLP-1 agent selection, dosing, and treatment adherence further restricts mechanistic inference.

Most cohorts were derived from large United States administrative databases, which limits generalisability to non-United States healthcare systems and prevents adjustment for important clinical variables such as glycaemic variability, inflammatory status, nutritional parameters, operative duration, surgeon volume, and institutional infection prevention protocols. Substantial statistical heterogeneity was observed in several analyses, particularly for total knee arthroplasty outcomes, and some pooled estimates were sensitive to single study influence. Several secondary medical outcomes were infrequent, limiting statistical power for safety assessment.

Follow-up durations ranging from 12 to 36 months were insufficient to evaluate long term implant survivorship, late periprosthetic joint infection, and mechanical failure. Restriction of the search to English-language publications may also have introduced language and publication bias. These limitations indicate that the observed associations should be interpreted as hypothesis-generating rather than causal and highlight the need for adequately powered prospective trials with standardised obesity definitions, clearly defined perioperative exposure windows, and long-term outcome assessment.

Future Directions

Adequately powered randomised trials, particularly in morbid obesity, should determine optimal timing, duration, and agent choice for perioperative GLP-1 therapy, include mechanistic endpoints (glycaemic variability, inflammatory biomarkers, wound-healing indices), and extend follow-up periods to assess PJI, readmission, revision, patient-reported outcomes, and cost-effectiveness. Harmonised definitions (e.g., International Consensus Meeting (ICM) criteria for PJI) and adherence capture are essential.

## Conclusions

Perioperative GLP-1 therapy in patients suffering from obesity undergoing arthroplasty is associated with moderate-certainty evidence of reduced PJI after THA and 90-day readmissions, with low-certainty evidence showing no clear effect on TKA-PJI or revision. These findings support GLP-1 receptor agonists as a promising component of perioperative optimisation, pending confirmation in future randomised trials and longer-term survivorship studies.
